# Supplementary material for: Patterns of Intron Gain and Loss in Fungi
Source: PLoS Biol. 2004 Nov 30;2(12):e422. doi: 10.1371/journal.pbio.0020422 (PMC532390; doi:10.1371/journal.pbio.0020422)
Supplement: Table S1 — Also available at http://genes.mit.edu/NielsenEtAl/. (4.3 MB ZIP). [file pbio.0020422.st001.zip › NielsenEtAl/html/595.html]

AN6288.1.NCU01605.1.MG03151.1.FG00301.1


```
 CLUSTAL W (1.82) Multiple Sequence Alignments - Introns Inserted


Sequence 1: NCU01605.1	222 aa
Sequence 2: FG00301.1	268 aa
Sequence 3: AN6288.1	218 aa
Sequence 4: MG03151.1	257 aa
Alignment Length: 279 aa
Number Identitical Residues: 167 aa
Alignment Score (without introns) 7216


MG03151.1 	MSKPSSNGGSAWPGSPSAMGTTIATPEIKIPDAVYKG-------------------QGLQ
NCU01605.1	------------------MSSFLT------------------------------------
FG00301.1 	-------MDSPYSPYTATMFQHLTPKSVSTMHQTNTAIIPAAISTKSLPLSDELSFADLE
AN6288.1  	------------------MASFITT-----------------------------------
          	                  *   ::.                                   

MG03151.1 	TARAR~NNRTRNQFRPRAAGKGGATSYQLRQYAEATLGGGSLRKVVKLPEGEDENEWLAV
NCU01605.1	---TV2NQRTRNQFRPRASGKGGATSYQLRQYAEATLGGGSLRKVVKLPEGEDENEWLAV
FG00301.1 	PLLNS~NQRTRNQFRPRV-GKGGSASYQLRQYAEVTLGGGSLRKVVKLPEGEDENEWLAV
AN6288.1  	----I2NARTRAPFKPRSAAKG-TTSYQLRQFAEATLGSGSLRKAVKLPEGEDLNEWLAV
          	      * ***  *:** :.** ::******:**.***.*****.******** ******

MG03151.1 	NM1VDFYNQINLLYGAITEFCSPQTCPEMKATDE2-~--FEYLWQDNENFKRPTKMAAPA
NCU01605.1	NM1VDFYNQINLLYGAITEFCSPQTCPEMKATDE~Y0FRFEYLWQDTENYKRPTKMPAPA
FG00301.1 	NM1VDFYNQINLLYGAITEFCSPISCPEMKATDE2-~--FEYLWQDNENFKRPTKMPAPA
AN6288.1  	NV1VDFYNQINLLYGAITEFCSPQTCPEMKATDE2-~--FEYLWQDSENYKRPTKMSAPE
          	*: ******************** :*********     *******.**:******.** 

MG03151.1 	YIEQLMAWVQGSIDNEQVLPSRIG~VPFPKTFPSLVRQIFKRMYRVYAHIYCHHYPVVRE
NCU01605.1	YIEQLMSWVQGNIDNEAVLPSRIG~VPFPKSFPALVRQIFKRMYRVYAHIYCHHYPVIRE
FG00301.1 	YIEQLMTWVQANIDNEQVLPSKIG1VPFPKSFPALVRQIFKRMYRVYAHIYCHHYPVIRE
AN6288.1  	YIEHLMSWVQGNIDNEQMFPSRLG1VPFPKAFSSLVRQIFKRMYRVYAHIYCHHYPVIVH
          	***:**:***..**** ::**::* *****:*.:***********************: .

MG03151.1 	LGLEPHLNTSFKQYVLFIDEHSLASGKDYWGPLGDLVESMLKSD
NCU01605.1	LGLEPHLNTSFKQYVLFIDEHNLATGKDFWGPLGDLVDSMLRSD
FG00301.1 	LGLEPHLNTSFKQYVLFVDEHSLASGRDYWGPLGDLVDSMLKSD
AN6288.1  	LGLEPHLNTSFKHYVLFIDEHRLASGKDFWGPLGDLVDSMLRSD
          	************:****:*** **:*:*:********:***:**
```
